# Determination of IR3535 in Topical Insect Repellents: A New HPLC‐DAD Analytical Approach and Compliance Assessment

**DOI:** 10.1002/bmc.70296

**Published:** 2025-12-09

**Authors:** Fernanda Fernandes Farias, Maria Cristina Santa Bárbara, Newton Andreo‐Filho, Patricia Santos Lopes, Mariana Sbaraglini Garcia Silva, Vanessa Cristina Martins Silva, Vânia Rodrigues Leite‐Silva

**Affiliations:** ^1^ Programa de Pós‐graduação em Medicina Translacional, Departamento de Medicina, Escola Paulista de Medicina Universidade Federal de São Paulo São Paulo Brazil; ^2^ Centro de Medicamentos, Cosméticos e Saneantes Instituto Adolfo Lutz São Paulo Brazil; ^3^ Departamento de Ciências Farmacêuticas Instituto de Ciências Ambientais, Químicas e Farmacêuticas, Universidade Federal de São Paulo (UNIFESP) Diadema Brazil; ^4^ Frazer Institute, Faculty of Medicine University of Queensland Brisbane Queensland Australia

**Keywords:** compliance, HPLC‐DAD, insect repellent, IR3535, validation

## Abstract

IR3535 is a synthetic active ingredient widely recognized for its efficacy in topical insect repellent formulations. Among commonly used repellent actives, it is distinguished by its favorable toxicological profile, making it suitable for use in children from 6 months of age. Ensuring the quality of insect repellents is a critical regulatory practice that directly contributes to public health protection. This study presents the development and validation of a novel, rapid 2.8‐min retention time, and robust high‐performance liquid chromatography method with diode array detection (HPLC‐DAD) for the quantification of IR3535 followed by its application to the analysis of six commercial topical formulations. The method was validated in accordance with ICH Q2(R2) guidelines, demonstrating excellent linearity (*R* ^2^ = 0.996), precision (RSD < 2%), recovery range (98.2%–101.4%), selectivity, low limits of quantification (0.01 mg/mL) and detection (0.003 mg/mL), as well as robustness. This analytical tool enables reliable monitoring of IR3535 content, ensuring product safety, efficacy, and ANVISA compliance. Only 2 out of 6 commercial products analyzed met specifications for IR3535 content. These findings underscore the importance of implementing rigorous quality control practices to ensure regulatory adherence and to protect consumer health and rights.

## 1 Introduction

The expansion of hot and humid regions due to climate change is fostering optimal conditions for mosquito proliferation and the global spread of vector‐borne viral diseases. A comprehensive approach that integrates preventive strategies aimed at reducing breeding sites, alongside the use of insect repellents, can be effective in minimizing mosquito bites and curbing disease transmission (Tavares et al. 2018).

A wide range of active ingredients is available in commercial repellents, making the choice important due to differences in concentration, duration of action, and cost, which can impact consumer health. Issues such as intoxication and skin absorption may arise if not properly selected. The active ingredient *N,N*‐diethyl‐3‐methylbenzamide (DEET), the most widely used repellent globally, is a synthetic chemical derived from a pesticide, but in high concentrations, it can be absorbed into the body, potentially causing allergic reactions like mucous irritation (Vilar et al. 2018).

**FIGURE 1:**
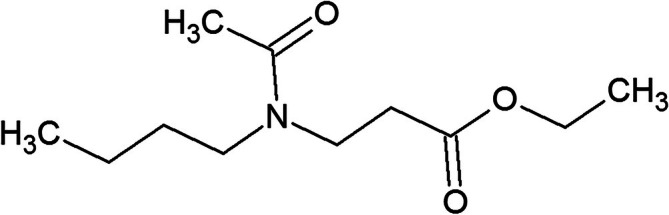
Molecular structure of IR3535.

In response to cases of intoxication with DEET, other repellents have been studied and explored, such as ethyl 3‐[acetyl(butyl)amino]propanoate (Insect Repellent 3535 [IR3535]), a compound derived from β‐alanine (Lupi et al. 2013). IR3535 is a colorless oily liquid, soluble in water and organic compounds such as methanol, isopropanol, and acetonitrile (LogP = 1.7 at 23°C). From a physicochemical perspective, IR3535 has the molecular formula C_11_H_21_NO_3_ (Figure 1), a molecular mass of 215.2893 g/mol, and a vapor pressure of 1.1 × 10^−3^ mmHg at 20°C (NCBI 2024).

Considered a highly effective substance, IR3535 was developed in 1975 by Merck & Co with the purpose of use in children; it is considered safe for use in children from 6 months of age (da Silva et al. 2018). In 2024, the Brazilian repellent market generated revenue of approximately USD 215.1 million, with projected growth to USD 315.4 million by 2030, representing a compound annual growth rate (CAGR) of 6.4%. (Grand View Research 2024).

While DEET remains the global gold standard, IR3535 offers lower toxicity and reduced irritation (Reynoso et al. 2017). However, there are reports in the literature that this compound can be absorbed through the skin and transferred to the blood and urine, at high concentrations (Lupi et al. 2013; Vilar et al. 2018).

The quality control of insect repellents is a critical regulatory measure that plays a key role in public health. It ensures that these products not only meet safety standards but also perform effectively in preventing insect bites, thereby reducing the risk of vector‐borne diseases. Rigorous testing and validation processes are essential to confirm that repellents contain the correct concentrations of active ingredients, are free from harmful contaminants, and do not pose any undue health risks to consumers, particularly in vulnerable populations such as children and pregnant women (Stefani et al. 2009). Maintaining consumer trust and safeguarding public health depends on rigorous quality control to ensure the reliability and effectiveness of these products.

Other methodologies have been reported for the determination of IR3535, including high‐performance liquid chromatography (HPLC) (Marselos and Archontaki 2002; Vilar et al. 2018), near infrared spectroscopy (NIR) (Vilar et al. 2020), and analyses in alternative matrices (Broschard et al. 2013). This study improves upon existing methods by achieving a 2.8‐min retention time, approximately 40% faster than prior reports.

The primary objective of the present work was to establish a reliable analytical tool for the quality control of insect repellent products, with particular emphasis on post‐marketing surveillance across different dosage forms. This focus is especially relevant, as routine quality monitoring of these products is not yet implemented in Brazil and many other countries. Accordingly, this study presents a novel HPLC method with diode array detection (HPLC‐DAD) that is simple, rapid, sensitive, and reproducible for the quantification of IR3535 in topical insect repellent formulations. The paper details the systematic process of method development, optimization, and validation, providing a robust framework for routine analytical and regulatory applications.

## 2 Material and Methods

### 2.1 Reagents and Chemicals

All reagents and solvents used were of analytical grade. The standard chemical material for IR3535 was donated by Merck, with 99.7% purity. A repellent sample in lotion presentation with the active ingredient at 15% was used for the validation phase (L1). After validation, the method was applied to two additional lotions (L2 and L3), one gel (G1), and two spray formulations (S1 and S2) (Table 1). With the exception of Spray (S2), all samples were commercially available products purchased from the market. Spray 2 is not included in the composition table, as it was a compounded (magistral) product. Its label stated only: “Repellent Merck IR3535 20% q.s. to 100 mL,” without specifying the excipients used. To prepare and solubilize the samples and standard, solvents such as isopropanol (Supelco) and acetonitrile (Sharlau) were used.

**TABLE 1:** Composition of the analyzed repellent products.

Function	Formulations composition				
	Lotion (L1)	Lotion (L2)	Lotion (L3)	Gel (G1)	Spray (S1)
Active ingredient	IR3535 (15.0% w/w)	IR3535 (15.0% w/w)	IR3535 (20.0% w/w)	IR3535 (15.0% w/w)	IR3535 (20.0% w/w)
Vehicle	Water	Water	Water	Water	Water
Surfactant	Polysorbate 20	Polysorbate 20	—	—	—
Emulsifier	Triethanolamine	Triethanolamine	—	Triethanolamine	—
		Stearic acid		PPG‐1‐PEG‐9 Lauryl glycol ether	
		Ceteareth 20			
		Propylene glycol stearate			
Viscosity/thickener agent	—	Cetearyl alcohol	Acrylate/C10‐30 alkyl acrylate crosspolymer	Acrylate/C10‐30 alkyl acrylate crosspolymer	—
		Hydroxiethyl cellulose	Cetearyl alcohol	Hydroxiethyl cellulose	
Preservative	Phenoxyethanol	Methylparaben	Phenoxyethanol	Phenoxyethanol	—
	Methylisothiazolinone	Ethylparaben	Methylisothiazolinone	Methylisothiazolinone	
		Propylparaben			
		Butylparaben			
		Phenoxyethanol			
Emollient	Glycerol	Glycerol	Glycerol	Propylene glycol	Propylene glycol
	Propylene glycol	Glyceryl stearate	Glyceryl stearate		*Aloe vera* leaf extract (extrato da folha de babosa)
	*Aloe barbadensis* extract (extrato de babosa)	*A. barbadensis* extract (extrato de babosa)	Cyclopentasiloxane		
		Dimethicone			
pH regulator	—	—	Isopropanolamine	—	—
Antioxidant	—	—	Butylated hydroxytoluene	—	—
Fragrance	Hydroxycitronellal	Linalol	—	—	—
	Benzyl salicylate	Farmesol			
	Linalol				
	Amyl cinnamal				
	Butylphenyl methylpropional				
Chelating agent	Disodium EDTA	Disodium EDTA	Tetrasodium EDTA	Disodium EDTA	—
Bittering agent	—	—	—	—	Denatonium benzoate

*Note:* Spray (S2) was a compounded product without excipient disclosure.

### 2.2 Equipment

The following equipment was used: Ultrasound Unique Ultrasonic Cleaner, GoldSun vacuum pump model 0411, and Mettler Toledo analytical balance model AL204. The water used to conduct all analysis was ultrapure, using the Purilab Classic system (18.2 MΩ/cm resistivity, Elga).

The analysis was performed on a model HPLC‐DAD (Agilent Technology, 1260 Infinity, Germany), equipped with an online degasser, binary pump (G1312B), autosampler (G1329B), column compartment with oven (G1316A), and diode array detector (DAD) (G1315D) system. The HPLC‐DAD data were processed by Open LAB CDS Chemstation Chromatographic Software (Version‐C.01.04).

### 2.3 Assay Method Conditions

The HPLC‐DAD parameters such as columns, detection wavelengths, flow rates, solvents, mobile phase compositions, injection volumes, and oven temperature were optimized to establish the most effective chromatographic conditions. Once optimal conditions were identified, the method underwent validation following the International Council for Harmonisation of Technical Requirements for Pharmaceuticals for Human Use (ICH) Q2(R2) guidelines for analytical procedures (ICH 2022). This included assessment of linearity, precision (repeatability and intermediate precision), accuracy, specificity, limit of quantification (LOQ), limit of detection (LOD), and robustness to confirm the method's reliability for its intended application.

### 2.4 Preparation of Sample Solution

For the validation process, a lotion‐based repellent sample (15% active ingredient) was prepared by weighing 650 mg into a 50 mL volumetric flask, diluting with isopropanol:water (50:50), and performing ultrasonic extraction. The solution was adjusted to volume with the diluent, followed by further dilution to a working concentration of 0.08 mg/mL using acetonitrile:water (50:50) as the mobile phase. Samples were filtered and transferred into vials for analysis. A similar procedure was applied to other formulations, including sprays and gels, adjusting the initial mass weighed based on the concentration of the active ingredient in each product to ensure a final concentration of 0.08 mg/mL.

### 2.5 Preparation of Standard Solution

The IR3535 standard solution was prepared by weighing 200 mg of the standard into a 100 mL volumetric flask, dissolving it with isopropanol:water (50:50), and adjusting the volume. A working concentration of 0.08 mg/mL was obtained via additional dilution in a 10 mL volumetric flask using the mobile phase. Standard solutions were filtered and stored in vials for analysis.

## 3 Results and Discussion

### 3.1 Assay Method Validation

Based on the extraction method described by Farias et al. (2025), the initial extraction of samples (lotion, gel, and spray) was tested using various solvents at 100% concentration, including acetonitrile, ethanol, and isopropanol. For most lotion samples, the resulting solution contained a considerable amount of particulate matter, suggesting insufficient extraction. Given that the lotion formulation was identified as the most complex for extraction, we selected it for validation parameter testing. The efficiency of IR3535 extraction from the repellent samples was assessed by comparing the results of samples extracted with 100% acetonitrile to those extracted with a 50:50 mixture of isopropanol and water. Because all tested samples were found to contain water in their formulations and the chromatographic peak area is proportional to the compound concentration, we noted that the peak areas for samples extracted with isopropanol and water were larger and more symmetrical compared to those extracted with acetonitrile alone.

System suitability tests were performed on the C18 and C8 columns. Changes in the mobile phase composition and temperature directly influenced the retention time, peak symmetry, tailing factor, and the number of theoretical plates, which is used to assess the efficiency of the chromatographic column.

During the establishment of chromatographic conditions, several tests were conducted using sample injections. A Poroshell C18 column with solid‐core packing, which typically enhances chromatographic peak efficiency, was initially tested. However, it failed to provide a stable baseline. Consequently, the column was replaced first with another C18 column and later with a C8 column. The tests performed with these two columns, varying the mobile phase ratio (ACN:H_2_O) and the column temperature, are presented in Table 2.

**TABLE 2:** System suitability tests performed on method optimization.

Column	Mobile phase ratio	Temperature (°C)	Retention time	Theoretical plates	Symmetry	Tailing factor
C18	ACN:H_2_O (50:50)	30°C	3.7 min	6946 6913	0.911 0.911	1.073 1.058
	ACN:H_2_O (60:40)	30°C	2.8 min	6466 6614	0.871 0.872	1.069 1.139
	ACN:H_2_O (60:40)	25°C	2.9 min	6200 5868	0.888 0.889	1.074 1.102
C8	ACN:H_2_O (60:40)	30°C	2.2 min	7231 7222	0.880 0.885	1.131 1.038
	ACN:H_2_O (60:40)	25°C	2.2 min	7489 7006	0.902 0.899	1.114 1.075
	ACN:H_2_O (50:50)	25°C	2.8 min	6922 7493	0.915 0.915	1.039 1.049
	^a^ ACN:H_2_O (50:50)	30°C	2.8 min	8088 8003	0.905 0.908	1.056 1.078

Abbreviations: ACN, acetonitrile; H_2_O, water.

^a^ Optimal conditions in system suitability tests, leading to the final chromatography conditions.

Based on the results presented in Table 2, the final condition, utilizing a C8 column at 30°C with a mobile phase composition of ACN:H_2_O (50:50) exhibited the highest theoretical plate counts and optimal peak symmetry. This indicates superior chromatographic efficiency, making it the selected condition for method validation.

**FIGURE 2:**
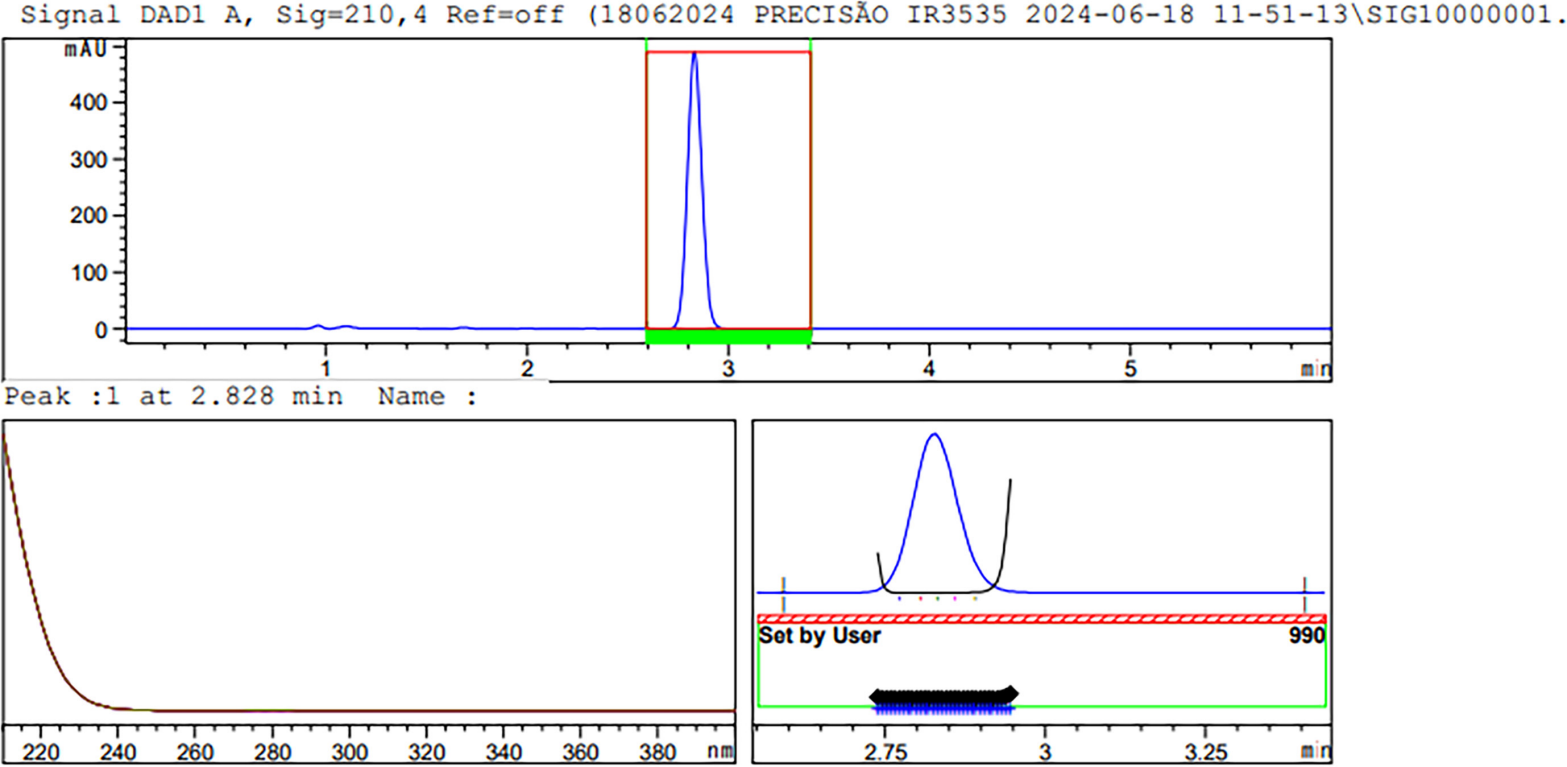
Peak purity factor, absorbance of IR3535, and threshold.

The chosen wavelength (210 nm) was based on the absorbance of the IR3535 compound, confirmed by peak purity within the established limit. The peak was obtained for IR3535 in a short run time, rapid 2.8‐min retention time (Figure 2). Other methods were reported in the literature involving the determination of the concentration of IR3535 in repellent samples. In the method developed by Vilar et al. (2018), the retention time of IR3535 was 4.78 min, while Marselos and Archontaki (2002) reported a retention time of less than 8.5 min. In contrast, the method in this study achieved a short retention time, significantly increasing the analytical throughput.

The standard was injected 6× with an injection volume of 20 μL and presented a relative standard deviation (RSD) of less than 2%.

The optimal chromatographic conditions established for the developed and validated HPLC‐DAD method were as follows: analyses were carried out using an HPLC system (1260 Infinity, Agilent Technologies) equipped with a DAD set at a detection wavelength of 210 nm. Separation was performed on an Avantor ACE C8 column (125 × 4.6 mm, 5 μm particle size), maintained at 30°C. The mobile phase consisted of acetonitrile and water (50:50, v/v), delivered at a flow rate of 1.0 mL/min, with an injection volume of 20 μL. These conditions resulted in enhanced chromatographic performance, including improved theoretical plate numbers, peak symmetry, and tailing factors, thereby supporting the selection of this method for routine analysis.

### 3.2 Validation Parameters

#### 3.2.1 Linearity

**FIGURE 3:**
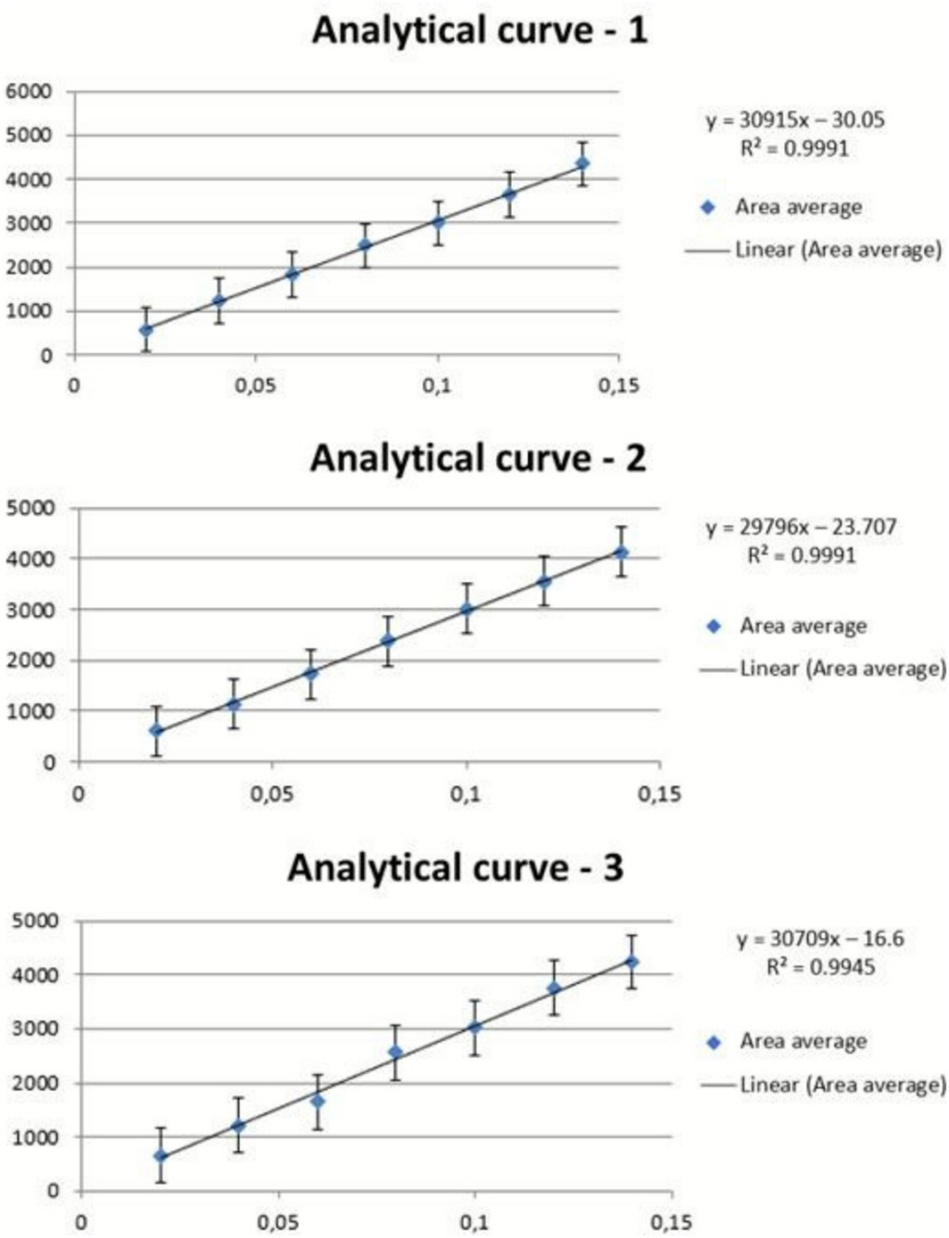
Analytical calibration curves for IR3535 quantification. Working concentration range of 0.02–0.14 mg/mL.

The linearity of an analytical procedure refers to its ability to produce results proportional to the analyte concentration within a specific range. In this study, the analytical curve was constructed with three analytical curves, within the working range: 0.02, 0.04, 0.06, 0.08, 0.1, 0.12, and 0.14 mg/mL (Figure 3); and the absence of outliers for each concentration level as well as the homoscedasticity of the data were confirmed. A coefficient of determination *R* ^2^ = 0.996 was obtained. Therefore, this parameter was considered compliant.

#### 3.2.2 Precision

Precision was evaluated through repeatability and intermediate precision, expressed by the RSD, with an acceptance criterion of RSD < 2.0%. Repeatability was assessed using the same measurement procedure, by the same analyst, on the same instrument, under identical environmental conditions. Intermediate precision was evaluated similarly, but with different days analysts. Independent repetitions were performed with six determinations of IR3535 at 0.08 mg/mL. The results showed an RSD of 1.54% for repeatability and 0.7% for intermediate precision (Table 3), thus meeting the established criteria for compliance.

**TABLE 3:** Results of repeatability and intermediate precision validation tests and *Q* test to the data.

Nominal concentration of IR3535 (%)	Intra‐day/repeatability		Inter‐day/intermediate precision	
	Concentration found (%)	RSD (%)	Concentration found (%)	RSD (%)
15	14.35	1.54	14.93	0.7
	13.79		14.77	
	14.44		14.83	
	14.06		15.07	
	14.26		14.95	
	14.27		14.79	

Intra‐day		Inter‐day	
Test *Q* with 95% confidence		Test *Q* with 95% confidence	
*Q* calculated (13.79)	0.415	*Q* calculated (14.77)	0.067
*Q* calculated (14.44)	0.138	*Q* calculated (15.07)	0.400
*Q* tabulated	0.560	*Q* tabulated	0.560

We applied the Dixon's *Q* test to check for the presence of outliers to precision data. Because the calculated *Q* values were lower than the critical value for *n* = 6 (0.560), it can be concluded that there are no outliers in the data obtained from the intra‐day and inter‐day assays.

#### 3.2.3 Accuracy

Accuracy by recovery study was made three times as a recommendation written on ICH (Q2)R2 (range: 98%–102%). The analyte recovery can be estimated by analyzing samples spiked with known amounts of the analyte. Samples may be fortified with the analyte at least three different concentrations (low, medium, and high) within the method's working range (0.06, 0.08, and 0.1 mg/mL). The recovery was adequate, as shown in Table 4.

**TABLE 4:** Accuracy assessment of IR3535 quantification by recovery study at three concentration levels according to ICH (Q2)R2 guidelines.

Added concentration of IR3535 standard (mg/mL)	Mean recovery (%)			Mean	RSD (%)
	1	2	3		
0.06	98.642	99.538	101.419	99.87	1.42
0.08	100.628	100.500	98.707	99.94	1.07
0.1	99.724	101.104	98.277	99.70	1.42

The recovery range reported by Vilar et al. (2018) was 94.7%–109.2%, whereas in this study, the recovery range was 98.2%–101.4%.

#### 3.2.4 Selectivity

Selectivity was evaluated by assessing matrix interference in the sample. Two analytical curves were prepared with the same analyte addition for each concentration level. One curve was prepared with the analyte added to the sample matrix (which already contains an analyte level), while the other analytical curve was prepared without the sample matrix. Five concentration levels were prepared and injected in sextuplicate. Standard concentrations were: 0.04, 0.06, 0.08, 0.1, and 0.12 mg/mL; for sample plus standard: 0.05, 0.07, 0.09, 0.11, and 0.13 mg/mL; and the sample alone at 0.01 mg/mL. The spectral homogeneity of a chromatographic peak indicates its chromatographic purity, which was satisfactorily demonstrated through assessment via OpenLab software.

#### 3.2.5 LOQ and LOD

The LOQ for an individual analytical procedure is the smallest amount of analyte in a sample that can be quantitatively determined with suitable precision and accuracy. The first point of the analytical curve (0.02 mg/mL) was tested, along with half of this concentration, both yielding an RSD below 2%, corresponding to an LOQ of 0.01 mg/mL for IR3535.

The LOD for an individual analytical procedure is the smallest amount of analyte that can be detected in a sample, though not necessarily quantified as an exact value. The LOD was calculated based on the LOQ by dividing the LOQ by 3.3, resulting in an LOD of 0.003 mg/mL.

#### 3.2.6 Robustness

Robustness was assessed using one‐way ANOVA. Deliberate variations in column oven temperature, flow rate, and detector wavelength were evaluated, as detailed in Table 5. The one‐way ANOVA results indicated no significant differences in the IR3535 content when comparing the validated method's chromatographic conditions to those under intentional variations. The calculated *F*‐value was lower than the critical *F*‐value, and the *p*‐value was greater than 0.05, demonstrating that the method is robust.

**TABLE 5:** Robustness assessment of IR3535 quantification method using one‐way ANOVA with deliberate variations in chromatographic conditions.

Chromatography conditions and its variations		*p* ^a^ (> 0.05)	*F* factor^a^ (less than *F* critical = 3.554557146)
Wavelength	210 ± 1 nm	0.999984	1.58219^−5^
Temperature	30 ± 1°C	0.450913	0.83278764
Flow	1.0 ± 0.1 mL/min	0.345481	1.128116156

^a^ Calculated by one‐way ANOVA.

Notably, neither Vilar et al. (2018) nor Marselos and Archontaki (2002) presented data on the robustness of their methods.

Table 6 presents the acceptance criteria for each evaluated parameter and the results obtained from the full validation of the active ingredient IR3535, demonstrating its satisfactory performance in all parameters conducted.

**TABLE 6:** Results of the validation parameter tests, according to ICH Q2(R2) guidelines.

Parameter	Acceptance criterion	Result
Linearity	*R* ^2^ > 0.99	*R* ^2^ = 0.996
Repeatability	RSD ≤ 2.0%	RSD = 1.54%
Intermediate precision	RSD ≤ 2.0%	RSD = 0.7%
Accuracy	98.0%–102.0%	Concentrations of 0.06, 0.08, and 0.1 mg/mL Ranging from 98.2% to 101.4%
Selectivity	Absence of matrix interference and chromatographic purity	Matrix did not interfere with the sample reading. Homogeneous slopes Pure peak
Limit of quantification	Lowest concentration that shows repeatability compliant	0.01 mg/mL
Limit of detection	Lowest amount of analyte that can be detected in a sample	0.003 mg/mL
Robustness	Variation in temperature, flow, and wavelength	One‐way ANOVA No significant differences on temperature, flow, and wavelength (*p*: > 0.05)

Abbreviations: *r* ^2^, coefficient of determination; RSD, relative standard deviation.

Most of the studies on IR3535 are related to efficacy tests and the comparison of IR3535 with other active ingredients used as insect repellents (Carroll 2008; Moreau et al. 2020; Naucke et al. 2006).

When it comes to chemical identification and quantification analysis of the active ingredient, there are few reports in the literature on IR3535. Differently from what is proposed in this study, the analytical method for identification and determination of IR3535 according to WHO specifications is based on gas chromatography using a flame ionization detector and internal standardization using methyl undecanoate (NCBI 2025). Another study by Lemes et al. (2024) proposed a rather unconventional technique for the determination of DEET, Icaridin, and IR3535 in insect repellents using excitation–emission matrix (EEM) fluorescence spectroscopy and multiway calibration (Lemes et al. 2024). Using near‐infrared (NIR) spectroscopy and multivariate calibration, Vilar et al. (2020) determined DEET and IR3535 in commercial repellents.

Other available methods refer to biological (urine, blood) (Broschard et al. 2013) or environmental samples (surface water, sediments, wastewater) (Chen et al. 2012), and are not directly comparable to cosmetic formulations.

The validation of analytical methods using HPLC‐DAD is still widely employed today due to cost‐effectiveness, availability in various research and testing laboratories, wide‐ranging knowledge, the need for quality control of products for market release and population safety (Gupta et al. 2024; Iqbal et al. 2024; Özgén et al. 2024; Yousaf et al. 2023).

Specifically concerning the analysis by liquid chromatography, Marselos and Archontaki (2002) proposed the determination of IR3535 in insect repellent semi‐solid products using HPLC with a UV–VIS detector and univariate calibration. In our study, we verified the application of the method for other formulations such as sprays and gels. Vilar et al. (2018) proposed the determination of DEET and IR3535 in repellents using HPLC‐DAD and univariate calibration. However, the method developed in this study offers significant advantages over previously published approaches. It enables efficient extraction of the active ingredient from the formulation, employs optimized chromatographic conditions and mobile phase composition, and achieves a rapid retention time of 2.8 min. In addition, the method demonstrated excellent recovery (98.2%–101.4%) and robustness—features not reported in earlier methods.

### 3.3 Application of the Method

The validated HPLC‐DAD method was applied to analyze different six commercial insect repellent formulations containing IR3535. As per National Health Surveillance Agency (ANVISA) guidelines (ANVISA 2018), the maximum permissible variation is 10% or less of the nominal value stated on the product label. Only 2 out of 6 commercial products analyzed met specifications for IR3535 content, as shown in Table 7.

**TABLE 7:** Description of contents and variation according to ANVISA specifications.

Formulation type	Content described	Content observed/deviation	Variation allowed	Assessment (compliant/not compliant)
Lotion (L1)	15.00%	14.27% (−4.9%)	13.50%–16.50%	Compliant
Lotion (L2)	15.00%	11.93% (−20.5%)	13.50%–16.50%	Not compliant
Lotion (L3)	20.00%	7.56% (−62.2%)	18.00%–22.00%	Not compliant
Gel (G1)	15.00%	14.23% (−5.1%)	13.50%–16.50%	Compliant
Spray (S1)	20.00%	7.62% (−61.9%)	18.00%–22.00%	Not compliant
Spray (S2)	20.00%	8.38% (−58.1%)	18.00%–22.00%	Not compliant

With the exception of sample L1, which was used for method validation, and the gel sample, the remaining formulations showed unsatisfactory results, with IR3535 content outside the specified range. These results highlight the critical importance of quality control for insect repellents available on the market. Four samples were found to be non‐compliant, with significantly lower active ingredient contents than those stated on the label. This discrepancy can compromise consumer safety by substantially reducing the effectiveness of the repellent and, consequently, its ability to protect against insect bites. Non‐compliant products may expose millions of Brazilian consumers to vector‐borne diseases, including emerging arboviruses.

Marselos and Archontaki (2002) analyzed six samples of a gel with different lot numbers, all containing a nominal IR3535 concentration of 8% (w/w). The results showed that all samples were within the specified content limits. Vilar et al. (2018) evaluated 21 commercial formulations containing IR3535 and found that a significant number of them had active ingredient levels below those declared on the label, underscoring the need for stricter monitoring of this market.

The robustness of the method was demonstrated through successful analyses under varying conditions. Its implementation not only ensures ANVISA compliance but also enhances consumer safety by maintaining product efficacy. This approach highlights the practical and operational advantages of the developed method, positioning it as an efficient and reliable alternative to more complex and resource‐intensive analytical techniques.

## 4 Conclusions

An efficient method using HPLC‐DAD for IR3535 quantification was developed and validated. The findings demonstrated that the method is highly specific and accurate. Moreover, the method uses a straightforward mobile phase and requires minimal sample preparation, making it suitable for quality control in the analysis of insect repellent formulations. Additionally, rapid 2.8‐min retention time and 98.2%–101.4% recovery range were observed. The method underwent systematic evaluation through experimental trials, confirming its applicability to various product forms and ensuring it meets the specific requirements for its intended use. Only 2 out of 6 products analyzed were in compliance with the specification for IR3535 content. Consequently, implementing quality control for repellents containing IR3535 supports compliance with ANVISA guidelines and safeguards consumer rights. Future studies should investigate degradation pathways in failing products.

## Author Contributions

**Fernanda Fernandes Farias:** conceptualization, investigation, methodology, writing – original draft, validation, software, writing – review and editing, resources. **Maria Cristina Santa Bárbara:** investigation, methodology, validation, writing – original draft. **Mariana Sbaraglini Garcia Silva:** formal analysis, methodology, validation, software, writing review and editing. **Vanessa Cristina Martins Silva:** writing – original draft, writing – review and editing. **Vânia Rodrigues Leite‐Silva:** conceptualization, project administration, writing review and editing, supervision, resources.

## Conflicts of Interest

The authors declare no conflicts of interest.

## Data Availability

The data that support the findings of this study are available within the article. The raw data of this study are available from the corresponding author upon reasonable request.
